# Supplementary material for: Comparative assessment of large language models for microbial phenotype assignment
Source: Genome Biol. 2026 Jul 23;27:235. doi: 10.1186/s13059-026-04207-7 (PMC13397958; doi:10.1186/s13059-026-04207-7)
Supplement: Supplementary file 1 — Additional file 1: Supplementary text, figures, and tables. File format: Portable Document Format, pdf. Title of data: Supplementary text, figures, and tables supporting the evaluation of large language models for microbial phenotype assignment. Description of data: This file contains the supplementary text, supplementary figures, and supplementary tables cited in the manuscript. [file 13059_2026_4207_MOESM1_ESM.pdf]

Comparative assessment of large language models for microbial phenotype assignment

Supplementary information

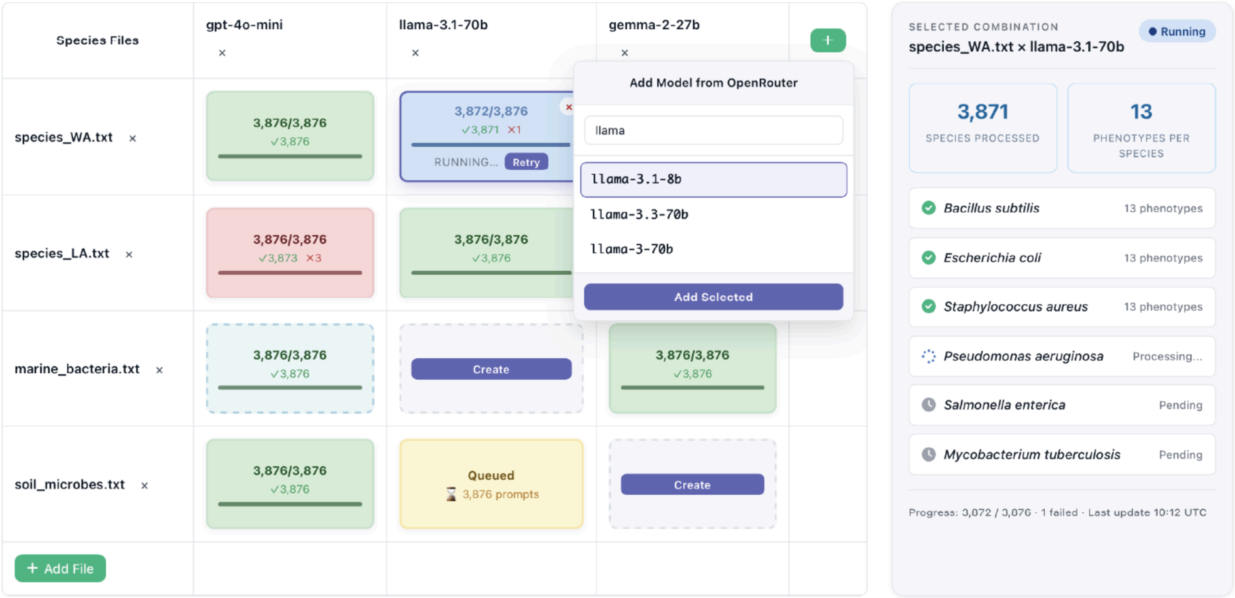

Fig S1: Admin Interface Overview. The pipeline provides a browser-based administrative interface for orchestrating large-scale phenotype assignment experiments. The interface enables systematic evaluation of multiple LLM models across diverse species collections, with real-time progress monitoring and automated job scheduling. Users can dynamically expand the model roster through OpenRouter integration (dropdown shown), track prediction status for each species-model combination, and access detailed extraction statistics including species processed, success rates, and failure counts for individual jobs.

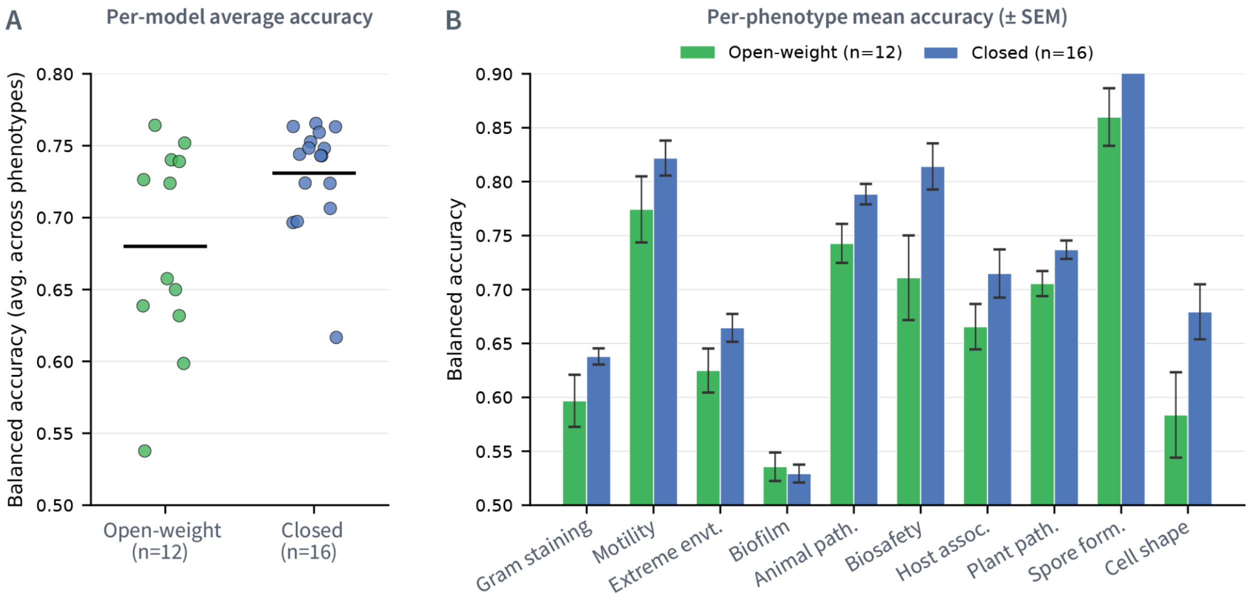

Fig S2: Phenotype prediction accuracy stratified by model availability. Balanced accuracy across phenotypic traits for open-weight versus closed-source models. Model availability was classified using the "Model accessibility" field from the Epoch.ai frontier-model metadata table: models labeled "Open weights" (unrestricted, restricted, or non-commercial) were treated as open-weight, and models labeled "API access" or "Hosted access (no API)" as closed-source. Of the 31 models evaluated on the phenotype benchmark, 28 could be classified (n = 12 open-weight, n = 16 closed-source); three models (gemini-pro, gpt-5, gpt-5-nano) lacked accessibility metadata in the reference table and were excluded. **A)** Per-model balanced accuracy averaged across the ten phenotypes; each dot is one model and the horizontal black bar marks the group mean. **B)** Per-phenotype mean balanced accuracy (± SEM across models) for each category. Top models in both categories achieve comparable performance, with the best open-weight models (DeepSeek-R1, 0.76; gpt-oss-120b, 0.75) within 0.2 percentage points of the strongest closed-source models (Gemini 2.5 Pro, 0.77; Claude 3.5 Sonnet and Grok-3-mini, 0.76).

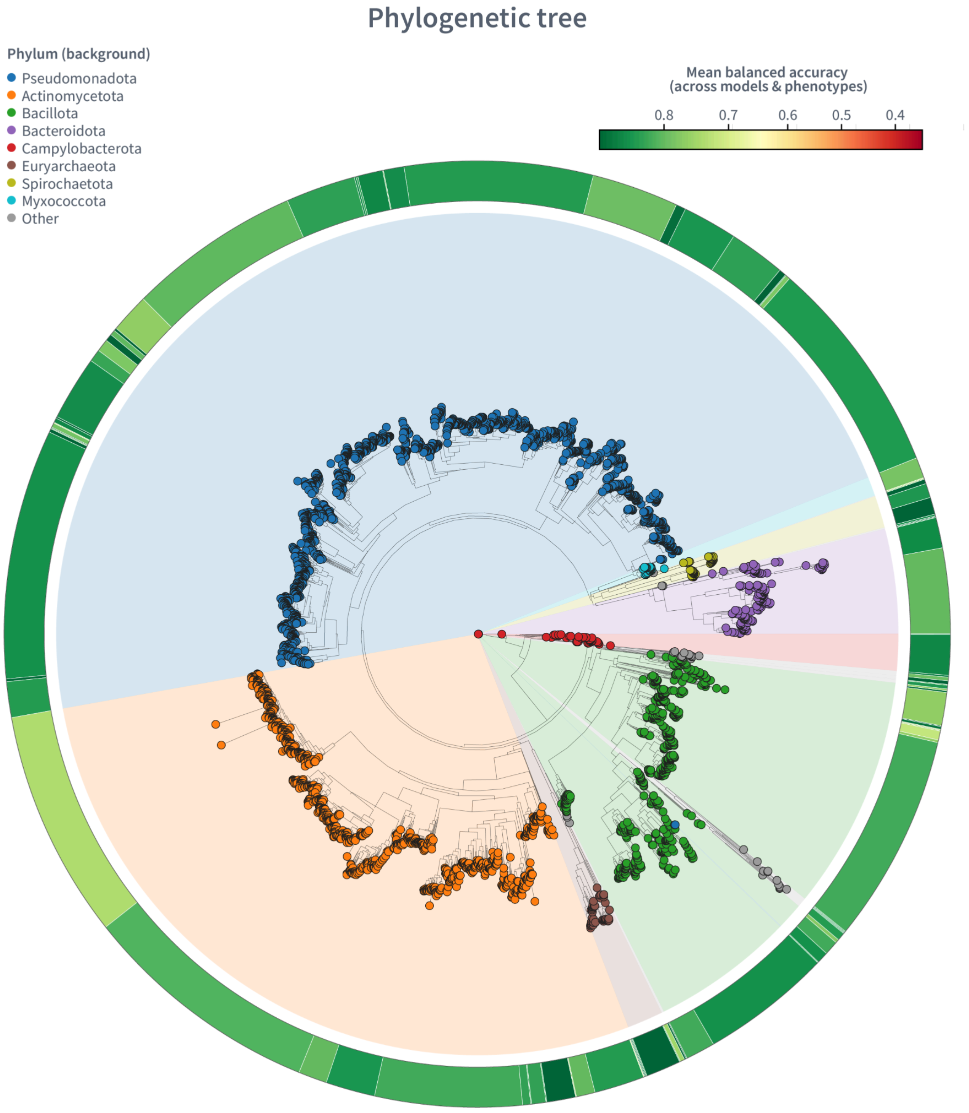

Fig S3: LM phenotype-prediction accuracy projected on a PhyloPhlAn phylogeny of the benchmark species. Circular phylogram of the 3,270 species from the WA evaluation subset that could be placed on a reference phylogeny built with PhyloPhlAn from a concatenated marker-gene alignment of the benchmark genomes. Each tip represents one species and is colored by its NCBI Phylum; the tinted background wedges show the same Phylum assignment. The outer ring shows the mean balanced accuracy of phenotype prediction, aggregated at the level of NCBI Order. For each Order, accuracy is computed as the mean per-species accuracy averaged across the ten evaluated phenotypes and all evaluated models. Species with fewer than three evaluated models are excluded from the aggregate. The diverging color scale is centered at the global mean (≈0.65) so that greener arcs indicate Orders where LLMs outperform the overall benchmark average and redder arcs indicate Orders where they underperform.

Table S1: Phenotypic and morphological attributes of microbial taxa used for LLM evaluation.The table shows the ten phenotypic traits evaluated across two data subsets: the

well-annotated (WA) subset with ≤5 missing values per species (n=3,876 species) and the low-assigned (LA) subset with sparse phenotypic coverage (n=15,256 species).

**Label**	**Type**	**Targets**	**Annotated fraction [in %]**	
			**WA subset**	**LA subset**
**Motility**	Binary	`[TRUE, FALSE]`	76.8	42.8
**Spore formation**	Binary	`[TRUE, FALSE]`	94	78.4
**Gram staining**	Multiclass	`[gram stain negative, gram stain positive, gram stain variable]`	99.8	93.2
**Cell shape**	Multiclass	`[bacillus, coccus, spirillum, tail]`	85.6	32.6
**Host association**	Binary	`[TRUE, FALSE]`	69.2	11.4
**Plant pathogenicity**	Binary	`[TRUE, FALSE]`	97.0	73.0
**Biosafety level**	Multiclass	`[biosafety level 1, biosafety level 2, biosafety level 3]`	96.5	84.2
**Extreme environment tolerance**	Binary	`[TRUE, FALSE]`	87.1	17.5
**Animal pathogenicity**	Binary	`[TRUE, FALSE]`	53.2	7.3
**Biofilm formation**	Binary	`[TRUE, FALSE]`	6.6	1.0

Table S2: Best-performing language models selected for individual microbial phenotype assignments. Each phenotype is paired with the language model achieving the highest balanced accuracy on species in the “Extensive” knowledge group within the WA dataset.

**Phenotype**	**Balanced accuracy**	**Best model (used for inference and knowledge grouping)**	**Sample size**
Spore formation	96.6%	Google gemini-2.5-pro	3,612
Cell shape	91.7%	OpenAI gpt-4.1-nano	3,290
Biosafety level	91.4%	Anthropic claude-3.5-sonnet	3,708
Motility	90.9%	OpenAI gpt-5	2,949
Animal pathogenicity	84.9%	Google gemini-flash-1.5	2,050
Host association	80.8%	OpenAI gpt-4o	2,665
Plant pathogenicity	79.4%	Google gemini-pro-1.5	3,723
Extreme environment tolerance	72.5%	DeepSeek deepseek-r1	3,350

Gram staining	69.5%	Google gemini-2.5-pro	3,840
Biofilm formation	61.7%	OpenAI gpt-4	253

Table S3: **Best-performing model selection for phenotype prediction using high-confidence filtering.** Best-performing model for each phenotype for species self-rated as having "Extensive" knowledge. Phenotypes where this filtering reduces accuracy (Motility, Extreme environment tolerance) are excluded.

**Phenotype**	**Model**	**Balanced Accuracy (%)**	**Samples**
Gram staining	xAI grok-3-mini	98.14	402
Animal pathogenicity	xAI grok-3-mini	86.14	327
Biosafety Level	xAI grok-3-mini	95.72	379
Host association	Anthropic claude-sonnet-4	83.99	259
Plant pathogenicity	Anthropic claude-sonnet-4	90.91	260
Spore formation	OpenAI gpt-oss-120b	99.07	351
Cell shape	OpenAI gpt-4.1-nano	93.31	658
